# Anethole Prevents the Alterations Produced by Diabetes Mellitus in the Sciatic Nerve of Rats

**DOI:** 10.3390/ijms25158133

**Published:** 2024-07-25

**Authors:** Bianca de Sousa Barbosa-Ferreira, Francisca Edilziane Rodrigues da Silva, Yuri de Abreu Gomes-Vasconcelos, Humberto Cavalcante Joca, Andrelina Noronha Coelho-de-Souza, Francisco Walber Ferreira-da-Silva, José Henrique Leal-Cardoso, Kerly Shamyra da Silva-Alves

**Affiliations:** 1 Laboratory of Electrophysiology, Superior Institute of Biomedical Sciences, State University of Ceará, Fortaleza 60.714-903, Ceará, Brazil; 2 Laboratory of Experimental Physiology, Superior Institute of Biomedical Sciences, State University of Ceará, Fortaleza 60.714-903, Ceará, Brazil; 3 Center of Exact Science and Technology, State University of Vale do Acaraú, Sobral 62.040-370, Ceará, Brazil

**Keywords:** anethole, diabetic neuropathy, sciatic nerve, compound action potential, nerve cross section, nerve conduction

## Abstract

Anethole is a terpenoid with antioxidant, anti-inflammatory, and neuronal blockade effects, and the present work was undertaken to study the neuroprotective activity of anethole against diabetes mellitus (DM)-induced neuropathy. Streptozotocin-induced DM rats were used to investigate the effects of anethole treatment on morphological, electrophysiological, and biochemical alterations of the sciatic nerve (SN). Anethole partially prevented the mechanical hyposensitivity caused by DM and fully prevented the DM-induced decrease in the cross-sectional area of the SN. In relation to electrophysiological properties of SN fibers, DM reduced the frequency of occurrence of the 3rd component of the compound action potential (CAP) by 15%. It also significantly reduced the conduction velocity of the 1st and 2nd CAP components from 104.6 ± 3.47 and 39.8 ± 1.02 to 89.9 ± 3.03 and 35.4 ± 1.56 m/s, respectively, and increased the duration of the 2nd CAP component from 0.66 ± 0.04 to 0.82 ± 0.09 ms. DM also increases oxidative stress in the SN, altering values related to thiol, TBARS, SOD, and CAT activities. Anethole was capable of fully preventing all these DM electrophysiological and biochemical alterations in the nerve. Thus, the magnitude of the DM-induced neural effects seen in this work, and the prevention afforded by anethole treatment, place this compound in a very favorable position as a potential therapeutic agent for treating diabetic peripheral neuropathy.

## 1. Introduction

Anethole is a terpenoid present as the principal constituent in over 20 species of plants, such as *Pimpinella anisum* L., *Foeniculum vulgare* var. dulce, and *Croton zehntneri* Pax et Hoffm [1]. Its pharmacological properties have been widely studied and its cicatricial [2], gastroprotective [3], nerve excitability inhibition [4,5], antinociceptive [6,7], antioxidant [3], and anti-inflammatory [8] effects strongly suggest anethole as a candidate for a therapeutic drug [1]. Moreover, the essential oil of *Croton zehntneri* (EOCz), whose major constituent is anethole (~80%), has been demonstrated to have similar pharmacological properties [2,4]. Furthermore, it was recently described that EOCz has beneficial pharmacological activity on diabetes mellitus (DM)-induced neuropathy [9,10].

Diabetic peripheral neuropathy (DPN) is a debilitating complication of DM, which is a chronic disease of high world incidence (about 10.5% of the population aged 20–79 years old) and alarming growth [11,12]. DPN affects more than 50% of patients with long-lasting DM [13], altering morphological and electrophysiological neuronal properties [14,15,16,17], leading to sensory and motor impairment [18]. DPN can affect any part of the body, but symptoms, such as tingling, burning, and pain, are initially noticed at the ends of the limbs [16,18]. Since the sciatic nerve (SN) innervates the lower limbs, it is widely used to study and understand the pathogenesis of DPN [19,20,21,22]. Moreover, the damage induced by DPN in the SN is related to the occurrence of diabetic foot, which can seriously progress to limb amputation [13,16,18]. Finally, the development of DPN can cause cardiovascular disorders and death [13].

Despite its severity, no specific treatment for DPN is currently available for clinical use; there are only attempts to prevent it through glycemic control and lifestyle modifications [11,13]. Furthermore, there are medications specifically used to treat neuropathic pain, such as anticonvulsants, antidepressants, and opioids. However, pain is only one symptom and does not always occur in all DPN. Even in patients with pain, the medication is not sufficiently effective [14,23]. Our research group has already investigated the activity of EOCz, rich in anethole, on DM-induced changes in ionic currents [10] and nerve excitability [9] and found its beneficial effects by normalizing the DPN-related alterations in those important electrophysiological parameters. Anethole itself possesses pharmacological properties such as inhibition of nerve excitability, antioxidant, and anti-inflammatory effects, which could act on several pathogenic mechanisms of DPN, differently to currently used medications. Thus, we hypothesize that anethole has a preventive activity against neural alterations induced by DM.

Due to the clinical and epidemiological importance of DPN, as well as the potential therapeutic relevance, low toxicity, and the fact that the effects of EOCz are similar but not identical to those of anethole [2,4], we investigated here the protective effect of anethole against DM-induced alterations in the morphology and electrophysiology of the peripheral nerve.

## 2. Results

### 2.1. Diabetic Model Characterization and Anethole Effects

**Figure 1:**
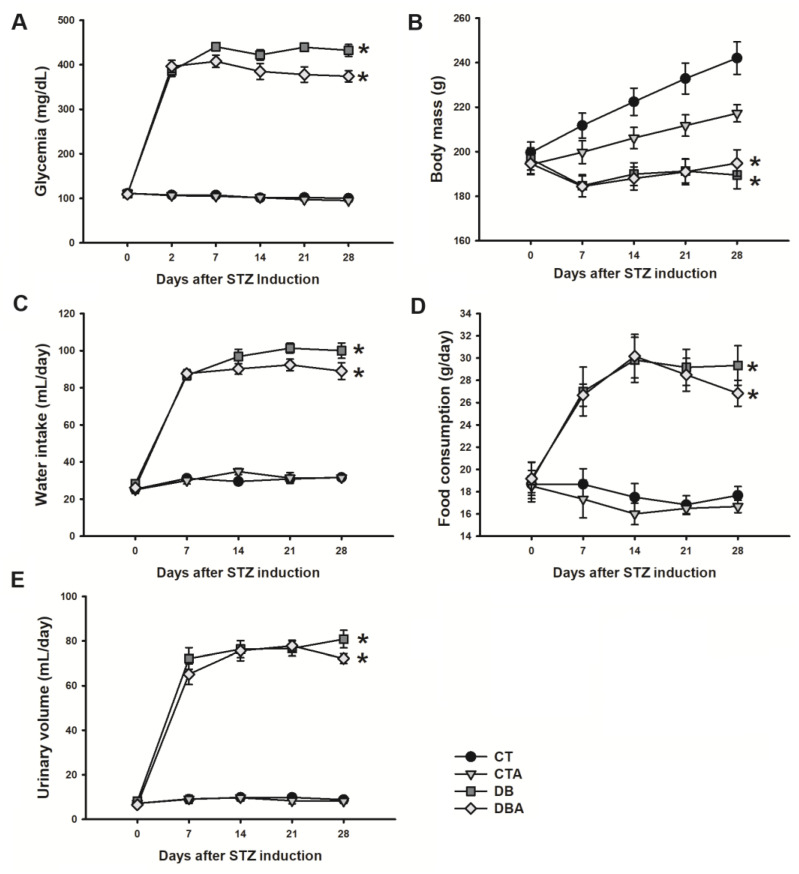
Characterization of the STZ-induced diabetes model and the effects of anethole. Panels (**A**,**B**) show the post-prandial concentration of blood glucose and body mass, respectively, of the control (CT; N = 37), anethole-treated control (CTA; N = 27), diabetic (DB; N = 32), and anethole-treated diabetic (DBA; N = 27) groups. Panels (**C**,**D**), and (**E**) show, respectively, water intake, food consumption, and urinary volume measured over 24 h (N = 6 for each group). The data are expressed as mean ± S.E.M and the * symbol demonstrates a statistically significant difference (*p* < 0.05) compared to values of the CT group; one-way ANOVA followed by the Dunn’s, Holm–Sidak or Tukey method (the most appropriate was used according to data characteristics).

In this work, the preventive effects of anethole on diabetic neuropathy were investigated in DM-induced rats. The animals were divided into control (CT), control treated with anethole (CTA), diabetic (DB), and diabetic treated with anethole (CTA) groups. Before DM induction with streptozotocin (STZ), all animals were euglycemic, and mean glycemic values were 111.0 ± 2.77 (n = 36), 111.5 ± 2.07 (n = 27), 110.3 ± 2.64 (n = 32), and 109.3 ± 2.34 (n = 27) mg/dL for the CT, CTA, DB, and DBA groups, respectively. After 48 h of STZ injection, the animals were hyperglycemic, and mean glycemic values were 386.0 ± 12.69 and 396.3 ± 13.43 mg/dL for the DB and DBA groups, respectively. In contrast, CT and CTA groups remained with glycemic levels of 107.5 ± 1.57 and 106.3 ± 1.97 mg/dL, respectively. The time course of glycemia in all groups remained stable during the observation period, but after week 2 (day 14), the DBA group displayed a reduced glycemia value compared to the DB group. At the end of 4th week, glycemia values were 100.0 ± 2.17, 94.9 ± 2.67, 432.3 ± 13.38, and 374.2 ± 12.72 mg/dL for the CT, CTA, DB, and DBA groups, respectively, and there was a statistically significant difference for DB and DBA versus CT (*p* < 0.05, ANOVA followed by Dunn’s post hoc method). The glycemia values are shown in Figure 1A.

Regarding body mass, all animal groups started with similar values (day 0) of 199.6 ± 4.69, 194.2 ± 4.52, 196.7 ± 4.95, and 194.7 ± 4.56 g for CT, CTA, DB, and DBA, respectively. CT and CTA animals gained mass during the DM observation period. Still, it was observed that there was a reduced rate of CTA mass increase and, at the end of week 4, the body mass of the CT and CTA groups were 242.0 ± 7.26 and 217.2 ± 3.88 g, respectively, and were significantly different (*p* = 0.009, one-way ANOVA followed by Holm–Sidak post hoc method). For the DM groups, no increase in body mass was found in the observation period and, at week 4, the DB and DBA mass values were 189.5 ± 6.10 and 194.9 ± 5.99 g, respectively, and there was a statistically significant difference between these values compared to the CT group (*p* ≤ 0.001; one-way ANOVA followed by Holm–Sidak post hoc method). Body mass values are shown in Figure 1B.

Concerning water intake, food consumption, and urinary volume, as shown in Figure 1C,D, the animals started with similar values on day 0, and there was a great increase in each parameter of the diabetic groups, which then remained stable during the observation period. The water intake, food consumption, and urinary volume in the DB group were 100.0 ± 4.08 mL/day, 29.3 ± 1.78 g/day, and 80.8 ± 3.94 mL/day, and presented similar values to the DBA group, which had values of 89.0 ± 4.47 mL/day, 26.8 ±1.17 g/day, and 72.2 ± 2.21 mL/day, respectively, with no statistically significant difference between these groups at week 4 (*p* > 0.05, one-way ANOVA). Also, control groups remained stable during the observation period and the CT group values of water intake, food consumption, and urinary volume were 31.7 ± 0.95 mL/day, 17.7 ± 0.80 g/day, and 8.8 ± 0.79 mL/day, and CTA group values were 31.5 ± 1.31 mL/day, 16.7 ± 0.56 g/day, and 8.2 ± 1.01 mL/day, respectively. There was a statistically significant difference between the DB and DBA groups compared to CT (*p* < 0.05, one-way ANOVA, followed by Tukey post hoc test).

### 2.2. Mechanical Sensitivity Test

**Figure 2:**
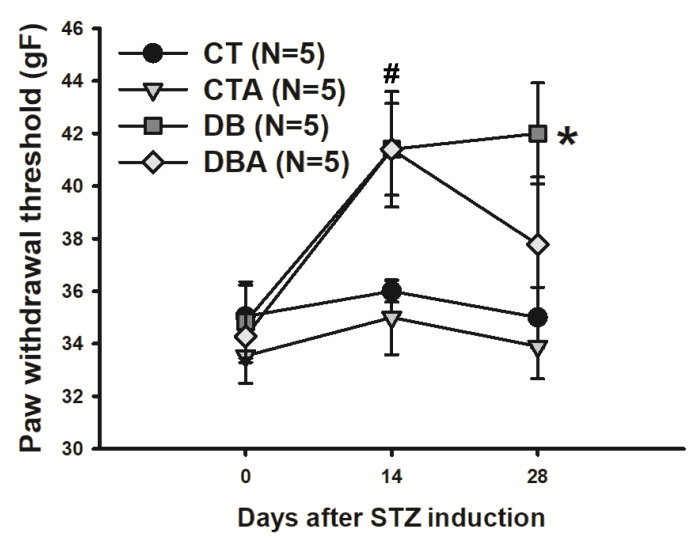
Effects of anethole treatment on mechanical sensitivity alterations induced by diabetes mellitus. CT, control; CTA, anethole-treated control; DB, diabetic; and DBA, anethole-treated diabetic. The data are expressed as mean ± S.E.M; (N) means number of animals per group. # *p* < 0.05 and * *p* < 0.05 compared to values of the CT group; one-way ANOVA followed by Tukey test.

The presence of diabetic neuropathy in animals can be characterized by changes in mechanical sensitivity [12,24]. Thus, this type of measurement was made before and during the DM time course. At day 0, the CT, CTA, DB, and DBA animals started with similar values (*p* > 0.05, one-way ANOVA) of 35.0 ± 1.32, 33.5 ± 1.05, 34.8 ± 1.39, and 34.2 ± 0.99 gF, respectively. At week 2 (day 14), the DB and DBA groups exhibited a significant (*p* = 0.004, one-way ANOVA followed by Tukey post hoc test) increase in paw withdrawal threshold compared to the CT group. This value remained significantly (*p* = 0.004, one-way ANOVA followed by Tukey test) higher and stable in the DB group (42.0 ± 1.92 gF) at the end of the DM period (day 28) compared to the CT and CTA groups (35.0 ±1.15 and 33.9 ± 1.23 gF, respectively). There was a tendency towards a reduction in paw threshold value in the DBA group (37.3 ± 2.56 gF), which at the 4th week was no longer different from the CT value. The sensitivity test data are shown in Figure 2.

### 2.3. Morphology and Morphometry of SN Medial Portion

**Figure 3:**
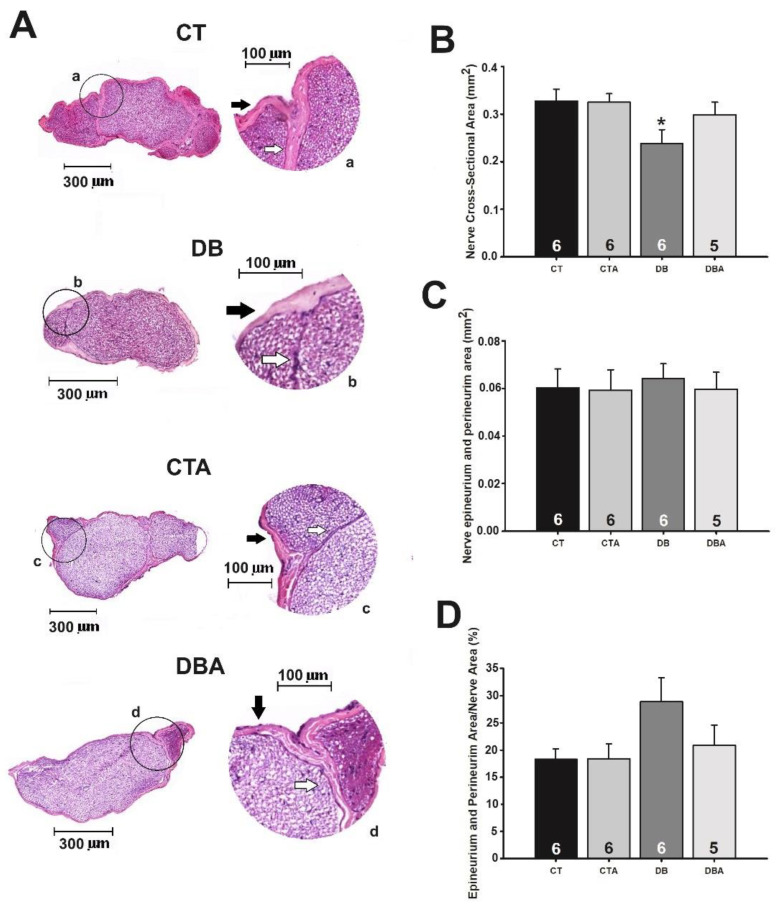
Effects of anethole treatment on morphological alterations induced by diabetes mellitus on the sciatic nerve of rats. Panel (**A**) shows a transverse cross-section of the medial portion of the sciatic nerve using a 10× objective. The lowercase letters (a–d) indicate insets and theirs respectively image magnifications. The black arrow indicates the epineurium, and the white arrow indicates the perineurium of the nerve. The calibration bar is displayed in each cross-section. Panels (**B**–**D**) show the mean ± S.E.M of the area of the nerve transverse section, the sum of epineurium and perineurium areas, and the proportion of epineurium and perineurium per area of the sciatic nerve, respectively. The same legend applies to all panels: CT, control; CTA, anethole-treated control; DB, diabetic; and DBA, anethole-treated diabetic. The number inside each bar indicates the number of nerves analyzed (n). * *p* < 0.05 compared to values of the CT group; one-way ANOVA followed by the Holm–Sidak post hoc method.

Once the induction of DM and its metabolic and mechanical sensitivity changes were demonstrated in rats, the occurrence of morphological modifications promoted by DM and the preventive effects of anethole on these neural alterations were studied. Figure 3, panel A, shows the microscope slides of the medial portion of the SN of the CT, CTA, DB, and DBA groups. As shown in Figure 3B, the DB group displayed a significant (*p* = 0.03, one-way ANOVA followed by Holm–Sidak post hoc test) reduction in the nerve cross-sectional area of the SN compared to the CT and CTA groups. The total cross-sectional area of the SN in CT, CTA, and DB were 0.327 ± 0.0251, 0.324 ± 0.0180, and 0.238 ± 0.0288 mm^2^, respectively. The DBA group showed a prevention by anethole against DM-induced alterations in the SN cross-sectional area since this value in this group was 0.298 ± 0.0271 mm^2^, which was not significantly different from the CT value.

Other morphometrics of the SN were altered by DM (Figure 3C,D). There was a tendency toward an increase (not significant, *p* > 0.05, one-way ANOVA) in the epineurium and perineurium area of the DB group compared to CT and a recovery toward control values in the DBA group. When the last parameter was normalized by the percentage of the nerve cross-sectional area, the mean values for CT, CTA, DB, and DBA groups were 18.2 ± 1.97, 18.4 ± 2.68, 28.8 ± 4.39, and 20.8 ± 3.74%, respectively.

### 2.4. Anethole Effects on Electrophysiological Changes Promoted by DM on Myelinated SN Fibers

Since DM decreased the SN cross-sectional area and anethole was able to fully prevent that, we investigated whether the same anethole protective effect happened with nerve electrophysiological parameters. Parameters related to the excitability and conductibility of the compound action potential (CAP) of the SN were employed and, this hypothesis was confirmed.

**Figure 4:**
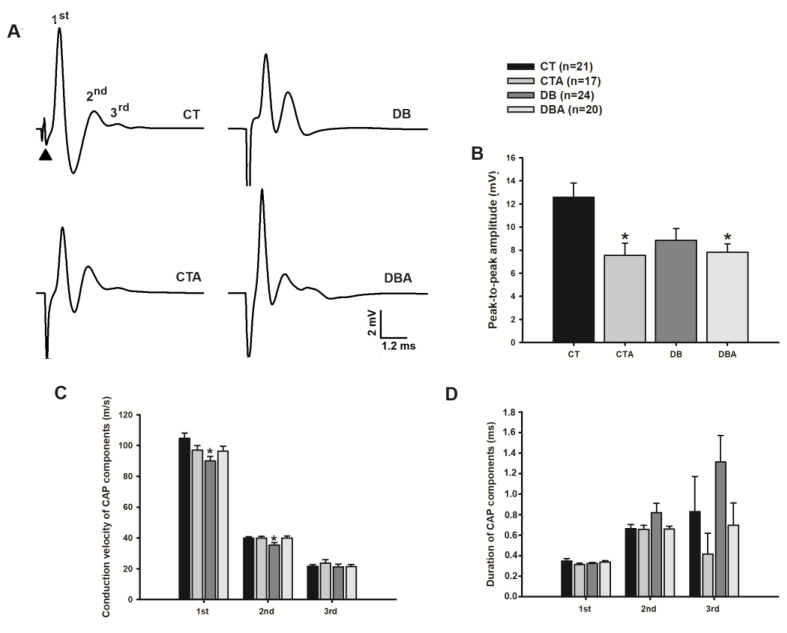
Effects of anethole treatment on electrophysiological alterations induced by diabetes mellitus on the sciatic nerve of rats. Panel (**A**) shows representative traces of the compound action potential (CAP) of the sciatic nerve. Each CAP component is indicated by an ordinal number, and the arrowhead indicates the stimulus artifact. The calibration bar is the same for all traces. Panels (**B**–**D**) show the peak-to-peak amplitude of the CAP, the conduction velocity, and duration at half-width of the CAP components, respectively. The same legend applies to all panels: CT, control; CTA, anethole-treated control; DB, diabetic; and DBA, anethole-treated diabetic. The data are expressed as mean ± E.PM, (n) means number of nerves per group, and * represents *p* < 0.05 compared to values of the CT group, one-way ANOVA followed by the Dunn’s or Holm–Sidak method.

The registered CAP commonly presented three waves, which we named the 1st, 2nd, and 3rd components, according to their order of appearance in the record (Figure 4A). It is worth mentioning that, differently to the other components, the 3rd component of myelinated fibers did not occur in all CAPs. The percentage of its occurrence was 72.7%, 70.0%, 57.9%, and 75.0% in CT, CTA, DB, and DBA, respectively. There was a tendency for a decrease in the occurrence of the 3rd component in the DB group (Figure 4A).

Concerning the CAP peak-to-peak amplitude (Figure 4B), the observed mean values were 12.6 ± 1.25, 7.6 ± 1.06, 8.8 ± 1.01, and 7.8 ± 0.71 mV for CT, CTA, DB, and DBA, respectively. This parameter was significantly decreased, as compared to control, in the CTA and DBA groups (*p* < 0.05, one-way ANOVA followed by Dunn’s method). Concerning the DB group, only a tendency toward a decrease was observed in the same comparison.

With respect to the velocity of conduction of the myelinated fibers, for the 1st, 2nd, and 3rd CAP components, the values were 104.6 ± 3.47, 39.8 ± 1.02, and 21.5 ± 1.31 m/s, respectively, for the CT group, while for DB groups they were 89.9 ± 3.03, 35.4 ± 1.56, and 21.1 ± 1.87 m/s; these DB group values for the 1st and 2nd components are significantly decreased when compared to the CT group (*p* < 0.05, one-way ANOVA followed by Holm–Sidak method; Figure 4C). Anethole did not significantly alter the velocity of conduction of any component of the CAP (CTA: 97.0 ± 2.95, 39.9 ± 1.16, and 23.7 ± 2.29 m/s for the 1st, 2nd, and 3rd component, respectively) and prevented the alterations caused by DM (DBA: 96.3 ± 3.28, 39.9 ± 1.27, and 21.3 ± 1.42 m/s, respectively, for the same components above; compare columns of CTA and DBA in Figure 4C).

Concerning the duration of the CAP components of the myelinated fibers, DM did not alter the duration of the 1st component (CT: 0.35 ± 0.02; DB: 0.33 ± 0.01 ms), while it increased it for the 2nd (CT: 0.66 ± 0.04; DB: 0.82 ± 0.09 ms) and 3rd (CT: 0.83 ± 0.34; DB: 1.31 ± 0.26 ms) components. As shown in Figure 4D, the anethole treatment did not alter the duration of any component of the CAP (CTA: 0.31 ± 0.01, 0.66 ± 0.04, and 0.42 ± 0.20 ms for the 1st, 2nd, and 3rd component, respectively) and prevented the alterations caused by DM (DBA: 0.34 ± 0.01, 0.66 ± 0.03, and 0.70 ± 0.22 ms for the 1st, 2nd, and 3rd component, respectively).

**Figure 5:**
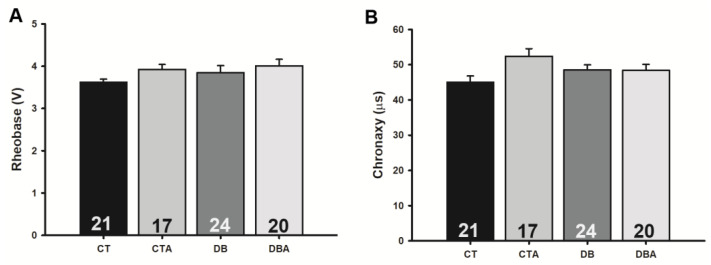
Effects of anethole treatment on the excitability of the sciatic nerve of control and diabetic rats. Panels (**A**,**B**) show the rheobase and chronaxy of the compound action potential (CAP) of the sciatic nerve of rats, respectively. The same legend applies to all panels: CT, control; CTA, anethole-treated control; DB, diabetic; and DBA, anethole-treated diabetic. The data are expressed as mean ± E.PM, and the number inside each bar (at the bottom) indicates the number of nerves evaluated (n). There were no statistically significant differences between groups (*p* > 0.05, one-way ANOVA).

Regarding excitability, a tendency to increase rheobase and chronaxy in CTA, DB, and DBA was observed, suggesting a decrease in excitability, but no significant alteration as compared to CT was observed (Figure 5).

### 2.5. Anethole Effects on Oxidative Stress Markers Promoted by DM on SN

**Figure 6:**
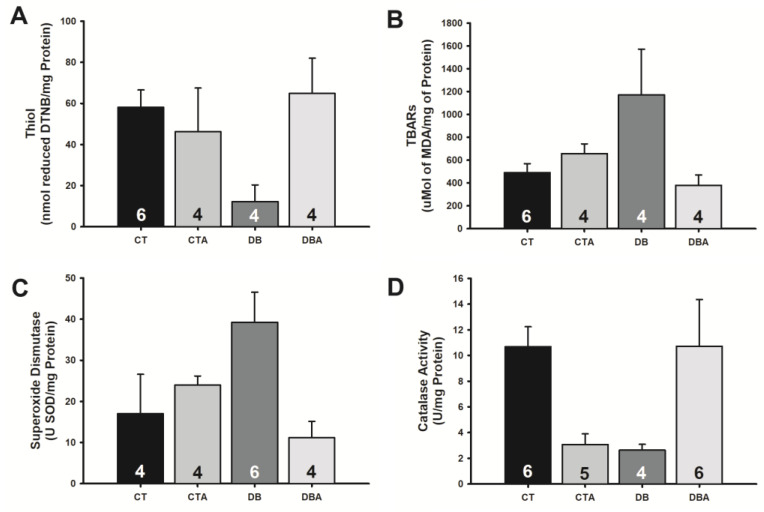
Effects of anethole treatment on oxidative stress induced by diabetes mellitus on the sciatic nerve of rats. Panels (**A**,**B**) are related to lipid peroxidation, demonstrating thiol and TBARS levels, respectively. Panels (**C**,**D**) show the activity of superoxide dismutase and catalase enzymes, respectively. The same legend applies to all panels: CT, control; CTA, anethole-treated control; DB, diabetic; and DBA, anethole-treated diabetic. The data are expressed as mean ± E.P.M, and the number inside each bar indicates the number of nerves evaluated (n). There were no statistically significant differences between groups (*p* > 0.05, one-way ANOVA).

DM induced marked alterations in the SN concentrations of thiol and thiobarbituric acid reactive substances (TBARS) and in the activity of superoxide dismutase (SOD) and catalase (CAT). Treatment of animals with anethole, in all cases, altered the values of oxidative stress markers to values closer to control than the respective values in the DB group (Figure 6). Thus, in the DB group, SN’s thiol concentration and catalase activity were decreased from 58.0 ± 8.46 and 10.7 ± 1.54 (CT values) to 12.2 ± 8.09 nmol reduced DTNB/mg of protein and 2.6 ± 0.45 U/mg of protein (DB values), respectively. In DM animals treated with anethole (DBA), these values returned to the proximity of control: 64.8 ± 17.14 nmol reduced DTNB/mg of protein and 10.7 ± 3.63 U/mg of protein (Figure 6A,D). Something similar occurred for TBARS concentrations (Figure 6B) and SOD activity (Figure 6C), except for the fact that DM increased the value of these parameters (CT: 490.7 ± 78.16 μmol MDA/mg of protein; 17.0 ± 9.60 U SOD/mg protein; DB: 1172.0 ± 400.00 μmol MDA/mg of protein; 39.2 ± 7.32 U SOD/mg protein; Figure 6B,C) and treatment of DM animals with anethole showed a tendency to normalize these values to control (DBA: 380.0 ± 89.62 μmol MDA/mg of protein; 11.1 ± 3.97 U SOD/mg protein).

## 3. Discussion

The major discovery of the present investigation is that the treatment of diabetic animals with anethole prevented morphological, electrophysiological, and oxidative stress abnormalities induced by DM in the SN, even in the presence of hyperglycemia. The magnitude of the preventive effect induced by anethole was relevant and, for some parameters, it nearly normalized them completely. Anethole has been the subject of studies by the scientific community due to its potential biological activities (low toxicity and pharmacological, mainly antioxidant and anti-inflammatory, effects). However, to the best of our knowledge, this is the first study investigating the effect of anethole on diabetic neuropathy.

These observations, although inferred from an experimental study, are also applicable to human DM. This is because the DM experimental model employed here (STZ-induced DM) is widely used for the study of diabetic neuropathy and reproduces symptoms, such as hyperglycemia, reduction in body mass, polydipsia, polyphagia, and polyuria, characteristics shared by both the experimental model [25] and human DM [26]. Anethole was not able to reverse the hyperglycemia caused by DM, but it showed a slight hypoglycemic effect. Other studies using anethole as a treatment for DM complications obtained similar hypoglycemic effects [27,28], and it has been suggested that this effect of small magnitude is due to anethole interference on key enzymes of carbohydrate metabolism [27].

Alterations in mechanical and thermal sensitivity are frequently observed in STZ-induced DM models and are related to the onset of diabetic neuropathy [29,30,31]. In the present study, we observed that diabetic animals developed mechanical hyposensitivity after 14 days of DM induction, suggesting the onset of diabetic neuropathy. Regarding anethole treatment, it partially reverted this hyposensitivity at the end of week 4, indicating a neuroprotective effect. Recently, it has been demonstrated that anethole prevented neuropathic pain induced by SN constriction in mice [7] and attenuated motor dysfunction in experimental Parkinson’s disease [32] attributed to neuroprotective mechanisms.

The SN is a component of the neural circuitry of the paw mechanical sensitivity [20] and it is a structure frequently affected by diabetic neuropathy [21,22]. Since significant alterations were observed here in mechanical sensitivity, the authors asked if these changes in function were also expressed at the level of nerve morphology. Like any other nerve, the SN is composed of axons individually wrapped by endoneurium. Groups of axons form fascicles, which are, in turn, enveloped by connective tissue called perineurium, and the collection of fascicles forms the nerve, which is surrounded by epineurium. Unlike axons, connective tissues do not perform signal processing and transmission functions [33]. A reduction in nerve cross-sectional area was observed in diabetic animals (DB group) and was strikingly prevented in animals treated with anethole (DBA group). This nerve atrophy occurred in the presence of increased connective tissue (epineurium and perineurium), suggesting that the reduction in the area seen in the DB group is unlikely to be due to a reduction in connective tissue. Thus, these data indicate changes in the axonal structure and/or a reduction in the number of axons. It is important to notice that the tendency toward an increase in the epineurium and perineurium areas was prevented by anethole treatment (Figure 3D, DBA group). Also, several histological studies showed that a reduction in the number of axons, demyelination, and axonal atrophy are common alterations in the nerves of diabetic animals induced by STZ [34,35,36,37].

The CAP is an electrically evoked response of nerve bundles and can present three components [38]. The CAP components are classified according to their chronological appearance in the recording as 1st, 2nd, and 3rd components, and each component represents a fiber population with well-defined conduction velocities [39,40]. In the present study, three CAP components were recorded and their conduction velocities are related to myelinated fibers, predominantly of the Aα (mean conduction velocity of 90 m/s, termed as 1st component), Aβ (39 m/s, termed as 2nd component), and Aδ (20 m/s, termed as 3rd component) types.

The presence of two CAP components in the SN is a common observation in the literature [4,9,14], which is not the case for the third component, which is more difficult to obtain as a stable signal component [38]. An interesting finding in this work is that DM showed a tendency to affect the 3rd CAP component, reducing its occurrence value by approximately 15%. The authors hypothesize that this reduction is related to degeneration and/or neuronal death of myelinated small fibers, since myelinated small diameters, such as Aδ fibers, are the first to be affected by diabetes [18,41]. Another hypothesis for this alteration is the dispersion of the conduction velocity of the 2nd component and fusion of the tail of this component with the 3rd one, making the 2nd component of long duration and the 3rd imperceptible. This velocity conduction dispersion is coherent with a desynchronization of nerve fibers, which promotes an increase in the duration of the 2nd component already cited in the vagus nerve of diabetic animals [9] and the sciatic nerve for a different diabetic model (n5-STZ [14]). Regarding anethole treatment, our data suggest that this treatment prevented the reduction of the 3rd component occurrence seen in the DB group, and this effect could be related to anethole’s antioxidant, anti-inflammatory, and neuroprotective effects [42,43].

Axonal degeneration and demyelination are consequences of diabetic neuropathy and impair neuronal functioning, leading to changes in nerve conductibility parameters such as prolonged latency, slower conduction velocities, and reduced CAP amplitudes [44]. In the present investigation, the DM reduced the peak-to-peak amplitude of CAP by 30% when compared to control data, corroborating with data found in the neonatal model of DM [14] and in mice [45]. Additionally, our study in the vagus nerve also showed a reduction in peak-to-peak CAP amplitude of myelinated fibers, but to a lesser extent [9].

Regarding anethole effects on the reduction of peak-to-peak amplitude in the DB group, it was observed that this constituent offered protection against this diabetic alteration since in the CTA and DBA groups, the CAP amplitudes have similar values (Figure 4B). Other investigations have shown that anethole acts by inhibiting neuronal activity through the blocking of voltage-dependent Na^+^ channels [4,5]. These data could suggest a possible action of anethole in neuronal excitability and the peak-to-peak amplitude reduction could also be related to the partial blockade of Na^+^ channels, thereby reducing the number of fibers contributing to the CAP signal.

In the present study, it was shown that DM reduced the conduction velocities of the 1st and 2nd CAP components of myelinated fibers by approximately 14%. A reduction in conduction velocity is the gold standard for the diagnosis of diabetic neuropathy, and similar magnitudes of reduction in the velocity of the CAP components in the sciatic nerve with diabetes have been documented [14,46,47,48].

Concerning anethole treatment, it completely prevented the reduction in conduction velocities of the 1st and 2nd CAP components, similar to what was observed for the vagus nerve of diabetic animals treated with the essential oil of *Croton zehntneri*, whose major component is anethole (80%), in which case the alteration prevention also amounted to 100% [9].

Nonetheless, there was no significant alteration in rheobase and chronaxy, parameters directly related to nerve excitability, but a slight change in those parameters. The tendency in alteration was also observed in other studies that found an increase in rheobase and chronaxy in the SN of rats [49] and an increase in chronaxy, but not in rheobase, in diabetic mice after 8 weeks of DM [45]. It seems that longer periods of diabetes are necessary for the change in excitability to fully progress.

The present investigation demonstrated that anethole possesses effects on diabetic neuropathy, the main complication of DM. Other protective effects of anethole in hepatic and renal complications induced by DM have been described and attributed to the anethole hypoglycemic effect [27,28]. However, as demonstrated in this work, the hypoglycemic effect is only partial and relatively small, and the glycemic value of the DB group treated with anethole is far from the glycemic value of the control group. Many studies highlight the pivotal role of inflammatory processes and oxidative stress in the pathogenesis of DPN [50,51]. Chronic hyperglycemia triggers inflammatory responses mediated by cytokines such as interleukin-6 (IL-6), tumor necrosis factor-alpha (TNF-alpha), and interleukin-1 beta (IL-1β), which contribute to neuroinflammation and subsequent nerve injury [51]. It has already been demonstrated that anethole has great anti-inflammatory activity, inhibiting various inflammatory mediators and pathways, such as cyclooxygenase enzymes, TNF-alpha, and IL-6 [52,53]. Additionally, the neuroprotective effect of anethole on the electrophysiology and morphology of mouse sciatic nerves with chronic constriction has been demonstrated, and the authors attribute this neuroprotection to anethole’s anti-inflammatory activity [7].

The present study did not investigate the participation of the anti-inflammatory activity of anethole but its participation in DM-induced oxidative stress, another pathway that disrupts the delicate balance between reactive oxygen species production and antioxidant defense mechanisms, leading to oxidative damage of nerve cells and exacerbating neuropathic symptoms [50,54]. It was shown that there was a decrease in thiols and catalase activities and an increase in SOD and TBARS levels in the diabetic group and that anethole treatment was also effective in restoring these parameters to control levels. Thus, we hypothesize that the neuroprotective effect of anethole is significantly related to its decrease in redox imbalance and does not largely depend on its slight hypoglycemic effect. In this scenario, we agree with others [3,8] who attribute importance to anethole’s antioxidant and anti-inflammatory effects in its protective mechanism of action. Nevertheless, other activities of anethole could participate in its mechanism of action, such as its local anesthetic activity [4,5].

Anethole is a natural compound recognized as safe by regulatory institutions and is already used in the food and cosmetic industries [55,56]. Although anethole is not yet used in the pharmaceutical industry as a medicine, it possesses properties that position it as a promising therapeutic agent. First, many studies have demonstrated its low toxicity in animal models and humans, with minimal adverse effects reported even at higher doses [55,56,57]. Cost-wise, anethole is economically viable due to its extraction from readily available plant sources and efficient manufacturing processes. Finally, the bioavailability of anethole can be variable depending on the formulation and route of administration. Anethole is slowly absorbed by the intestinal route and slowly metabolized but is effective when administered orally [55,56,57].

The dose of anethole (300 mg/kg) used here was chosen based on our previous studies about the effects of EOCz on DPN [9,10]. Although this dose could be considered high, its toxicity is considered low [8] since rats treated with 250 mg/kg anethole or EOCz for 70 days did not present behavior disturbances or changes in plasma biochemical parameters [52]. It is important to note that anethole is effective anti-inflammatory in doses (3–30 mg/kg) lower than used in this work, and a maximum anti-inflammatory effect may be reached at 30 mg/kg [8]. Thus, it is possible that the same magnitude of prevention demonstrated here could be achieved with lower doses of anethole.

Some limitations warrant consideration in our research. Firstly, we used rats and a DM model that causes a rapid development of hyperglycemia (within 48 h of induction), which may not fully translate to human physiological responses. Furthermore, the sample size in our biochemical assays was small, potentially limiting the robustness and generalizability of our results. Finally, we lacked a positive control for anethole, which could have provided comparative insights into its efficacy. It is worth mentioning, however, that there is no positive control for anethole in this case since there is no treatment for neuropathic alterations independent of glycemia correction, except for symptomatic treatment, like analgesics for pain. Looking forward, future investigations that explore, for example, myeloperoxidase activity and inflammatory infiltrates (as part of the inflammatory process), as well as the specification of neural components affected in DM (axon vs. myelin vs. connective tissues), can be undertaken to enhance our understanding of the therapeutic potential of anethole and its mechanisms of action.

## 4. Materials and Methods

### 4.1. Animals, Diabetes Mellitus Induction, and Anethole Treatment

All experimental protocols were approved by the Ethics Committee for the Use of Experimental Animals of the State University of Ceará (CEUA/UECE—protocol nº 02954540/2022). For this study, 122 *Wistar* rats (150–220 g, 8th week of life) of both sexes, provided by the Vivarium of the Institute of Biomedical Sciences—UECE and adequately housed and maintained at a controlled temperature (26 ± 2 °C) with a light/dark cycle (12/12 h) and free access to water and food, were used.

The experimental DM-induction was previously described [15]. Briefly, the animals were fasted for 8 h and randomly divided into CT, CTA, DB, and DBA groups. The animals of the DB and DBA groups received a single intraperitoneal injection of STZ (65 mg/kg) diluted in sodium citrate solution (0.1 M, pH 4.5, 4–8 °C). The CT and CTA groups did not receive STZ but only sodium citrate solution in an equivalent volume. The glycemia was measured after 48 h of DM-induction, and only animals with glycemia greater than 200 mg/dL were considered diabetic.

The anethole treatment also began after 48 h of DM induction and was performed daily for 4 weeks. All animals in the CTA and DBA groups received orally 300 mg/kg of trans-anethole (99% purity; Sigma Aldrich, St Louise, MO, USA) diluted in water. To ensure similar stress conditions, animals in groups CT and DB received only water in the same proportion of body mass.

The glycemia, body mass, urinary volume (24 h), water intake, and food consumption (24 h) were measured before (day 0) and weekly (days 7, 14, 21, and 28) after DM induction. All animals were euthanized at the end of 4 weeks of DM induction (12th week of animal life), and the SN was dissected for use in various experimental procedures, as described in the following sections.

### 4.2. Evaluation of Mechanical Sensitivity

Since sensitivity alteration indicates diabetic neuropathy, we performed a mechanical sensitivity test with rigid filament (Von Frey test). After the animals’ adaptation for 30 min in individual acrylic boxes (0.12 m × 0. 20 m × 0.17 m), a continuous mechanical stimulus (0.5 mm^2^ polypropylene tip) was applied perpendicularly to the plantar surface of the animal’s right hind paw until the animal completely removed this paw from the lower grid. The pressure applied was measured using a digital analgesimeter (IITC Inc., Life Science Instruments, Woodland Hills, CA, USA) and expressed in grams-force (gF). The application of the stimulus was repeated 5 times, with a 1 min interval between them. The average of these values was calculated and corresponded to the animal’s hind paw mechanical threshold. The first measurement was performed on the day before DM induction (day 0) and, after confirmation of DM, every 2 weeks until euthanasia (days 14 and 28).

### 4.3. Tissue Histological Preparation

The SN was fixed in a Carnoy solution at 4 °C containing 3:1 ethylic alcohol and glacial acetic acid. After that, the tissues were immersed in an increasing series of alcohol solutions (70% until 100%; 1 h in each concentration) and then in xylol 100% (2 series, 1 h each). After that, tissues were embedded in paraffin wax. Transversal cuts of 5 μm thickness in paraffin-embedded tissue were cut, mounted in microscope slides, and stained with hematoxylin and eosin.

### 4.4. Morphological and Morphometric Analysis

The nerve cross-sectional area of the medial portion was analyzed in a photomicroscope (Nikon E200; Tokyo, Japan) at 10× objective. The slide images were digitized with a high-resolution camera (Nikon Coolpix 4500, Tokyo, Japan) coupled to a computer. Images from the total nerve area and the epineurium and perineurium areas were analyzed using tools for images available in the ImageJ software (1.8.0 version). We also calculated the ratio between the epineurium and perineurium areas and total nerve area to verify a correlation between nerve area and peripheral connective tissue.

### 4.5. Electrophysiology

The technique of extracellular recording of the CAP was used to evaluate the conductibility and excitability of fibers present in the SN, following protocols previously described [38]. The modified Locke solution (composition (mM): NaCl 140, KCl 5.6, CaCl_2_ 2.2, MgCl_2_ 1.2, Tris(hydroxymethyl)aminomethane 10, and glucose 10; pH adjusted to 7.40 ± 0.01) was used for dissection and nutrition of the SN.

Briefly, the right SN was placed over the recording chamber electrodes, forming a loop immersed in Locke’s solution to keep the tissue viable throughout the experiment. The proximal region of the nerve was stimulated bilaterally with square wave pulses of 40 V amplitude, 0.1 ms duration, and delivered at a frequency of 0.2 Hz. The stimulus was generated by a stimulator (model S48, Grass Instruments, Nevada City, CA, USA) coupled with a stimulus isolator unit (model SIU 4678, Grass Instruments, Nevada City, CA, USA). This stimulation results in a depolarization of the membranes of the myelinated axons of the SN, which generate action potentials, which, after propagating, reach the distal end of the nerve and are recorded as CAP by a pair of electrodes. Subsequently, this analog signal was magnified by 1000 × and monitored in real-time using an oscilloscope (model TDS 340A, Tektronix, Beaverton, OR, USA), and its conversion was carried out using an A/D interface (Digidata 1322 A, Axon Instruments, San Jose, CA, USA). Finally, the Axoscope software (Axon Instruments, Inc., 10.0 version) was used for data recording and analysis. The CAP recording went through a stabilization period of 90 min, the time necessary for the peak-to-peak amplitude to become stable, so that the conductibility and excitability measurements could be evaluated.

Regarding the evaluation of CAP conductibility, the following parameters were used: (1) peak-to-peak amplitude, calculated by the sum (absolute values) of the largest positive amplitude with the largest negative amplitude; (2) conduction velocity of the CAP components, calculated by dividing the space covered by the CAP and the time interval between the beginning of the stimulus and the peak of each component; and (3) CAP component duration, which was measured at 50% of the positive amplitude of each component. Rheobase and chronaxy were used to measure the excitability of SN. The rheobase is the minimum pulse amplitude necessary to evoke a CAP when the nerve is stimulated by a very long pulse (1.0 ms). Chronaxy represents the minimum pulse duration to obtain a nervous response when the nerve is stimulated with twice the rheobase value. Rheobase and chronaxy are very important measures of the excitability of electrically excitable cells because they offer a resource to quantify the excitability in response to electrical stimulation precisely. Excitability is a major property of neural cells. Chronaxy differs from rheobase because it depends on cell membrane capacitance, while rheobase does not.

### 4.6. Sciatic Nerve Oxidative Stress Analysis

The oxidative damage promoted by DPN was evaluated by measuring the lipid peroxidation and thiol levels as damage markers and SOD and CAT enzyme activities as protective markers of SN.

SN was homogenized, with a Potter-type homogenizer (Fisatom, model 713DS, Perdizes, Brazil), in 900 μL of Tris-buffer containing Tris-HCl (10 mM), NaCl (0.9% *v*/*v*), phenylmethylsulphonyl (1 mM), and aprotinin (0.5 µM/mL), pH adjusted to 7.40. The homogenates were centrifuged at 720 g for 10 min (4 °C), and the supernatant was collected and stored at −80 °C until assay measurements. All damage or protective markers were normalized by the total protein content determined using the Lowry method [58]. For CAT activity, 10 µL of supernatant was mixed with phosphate saline buffer containing 0.053% oxygen peroxide. The time for the reaction was set to 60 s at 25 °C, and absorbance was read in a spectrophotometer (240 nm). CAT activity was expressed as H_2_O_2_ U/mg of protein [59]. Regarding SOD activity, the inhibition of auto-oxidation of adrenaline was evaluated during the 3 min after the start of the reaction. The SOD activity (U) was defined as the quantity necessary to inhibit 50% of adrenaline auto-oxidation. The absorbance was read in a spectrophotometer (480 nm), and enzyme activity was expressed as U/mg of protein [60].

The thiol levels were measured by a decrease in dithionitrobenzoic acid (DNTB) in the presence of the sulfhydryl group (-SH). A volume of 20 μL of supernatant was mixed with 150 μL of buffer solution (200 mM Tris and 20 mM EDTA), 820 μL methanol, and 10 μL of DNTB (10 mM). Afterward, the reaction solution was incubated at room temperature for 15 min and centrifugated for 15 min at 3000× *g* and 25 °C. The absorbance of the supernatant was measured with a spectrophotometer (412 nm), and the thiol quantity was expressed as nmol of DNTB/mg of protein [61]. The measurement of thiobarbituric acid reactive substances (TBARS) was made as follows: 200 µL of 10% trichloroacetic acid (TCA) was added to 200 µL of supernatant and centrifuged for 15 min at 2200× *g*. After that, 200 µL of this solution was added to TCA, incubated at 95 °C for 10 min, and absorbance measured in a spectrophotometer (535 nm). The TBARS levels were compared to the malondialdehyde standard curve and expressed as nmol/mg of protein [62].

### 4.7. Statistical Analysis

All statistical analysis and graphics were made in Sigmaplot (14.5 version), and data are expressed as mean ± S.E.M. The total number of experiments was represented by “n”, and a comparison between groups was made using one-way ANOVAs followed by appropriate post hoc tests. *p* < 0.05 indicates a significant statistical difference.

## 5. Conclusions

DM causes several abnormalities in the peripheral nervous system that impair nerve function. The great degree of preventive activity offered by anethole on DPN, without correction of hyperglycemia, showed that mechanisms other than glycemia correction are available for treating neuropathy, at least as a complementary treatment. This preventive activity, coupled with the low toxicity of this terpenoid, places anethole in a very favorable position as a potential therapeutic agent for the treatment of diabetic peripheral neuropathy.

## Data Availability

The data in this article can be made available upon request.
